# Insights from Germany’s Largest Diabetes Camp for Young People: Who Attends, Why, and What Do They Gain?

**DOI:** 10.3390/children13101386

**Published:** 2026-10-12

**Authors:** Su-Jong Kim-Dorner, Henrike Fritsch, Karin Lange, Gundula Ernst

**Affiliations:** Department of Medical Psychology, Hannover Medical School, Carl Neuberg-Str. 1, 30625 Hannover, Germany; kim-dorner.su-jong@mh-hannover.de (S.-J.K.-D.); lange.karin@mh-hannover.de (K.L.)

**Keywords:** young people, complementary care, type 1 diabetes, psychological well-being, youth camps

## Abstract

**Highlights:**

**What are the main findings?**

- Participants were predominantly highly educated females, with social connection identified as the primary motivation for participation and diabetes technologies as the primary area of interest.
- Participants reported improvements in diabetes self-management and mental well-being, alongside reduction in diabetes distress.

**What are the implications of the main findings?**

- Participants’ motivations and interests provide practical guidance for designing camp programs that align with the needs of AYA; however, the overrepresentation of highly educated females without a migration background highlights the need for broader outreach.
- As a complement to clinical care, youth camps may support empowerment, enhance diabetes self-management, and reduce diabetes-related distress in AYA.

**Abstract:**

**Background:** Adolescents and young adults (AYA) with type 1 diabetes face unique challenges in diabetes management due to hormonal fluctuations, lifestyle changes, and the transition to adult healthcare. Despite this, participation in traditional diabetes education courses is low. Diabetes camps may help address this gap by combining education, peer interaction, and psychosocial support. This study aimed to characterize AYA attending a diabetes camp, explore their motivations and interests, and evaluate associations between camp participation, psychosocial outcomes, and HbA1c. **Methods:** In this longitudinal study, participants completed online surveys before and three months after the camp. Data included demographic and clinical information, motivations for attending, topic interests, and satisfaction. Standardized measures included PAID-5 (diabetes-related stress), WHO-5 (mental well-being), and GAD-7 (anxiety). **Results:** Of 239 registered individuals, 178 completed the baseline survey (mean age 21.6 (±3.5) years, diabetes duration 10.8 (±5.9) years, 69.7% female, 17.4% migrant background, mean HbA1c 7.43% (±1.08)). Social interaction and interest in modern therapies were key motivations for participation. At follow-up, participants (n = 71) reported high satisfaction, with 74.3% indicating improved motivation for diabetes management, 64.3% improved self-management, and 58.6% greater self-confidence. Diabetes distress decreased and well-being improved (both *p* = 0.01), while anxiety and HbA1c remained unchanged. **Conclusions:** Diabetes camps may represent a valuable complement to outpatient care for AYA. Participation was associated with lower diabetes-related distress and improved well-being and may support self-management and self-confidence while addressing psychosocial and educational needs. However, as participants were predominantly highly educated females without a migration background, greater efforts are needed to engage a more diverse population.

## 1. Introduction

Type 1 diabetes (T1D) is the most prevalent metabolic disorder among children and adolescents. Effective management requires continuous glucose monitoring and regulation, which can be physically and psychologically demanding. Adolescents and young adults (AYA) often struggle to balance age-appropriate lifestyles with the demands of diabetes management.

This period also involves the transition from pediatric to adult care, during which many AYA have difficulty assuming full responsibility for self-management [1]. They are often unfamiliar with how stress, sleep, physical exertion, and alcohol affect metabolism and insulin dosing [1,2]. Growing awareness of the chronic nature of T1D and its long-term consequences adds to this burden. These challenges are frequently reflected in suboptimal metabolic control and elevated psychological distress among AYA [3]. Moreover, many AYA lack access to psychological counseling or peer networks, leaving them without opportunities to share concerns in a supportive environment.

Diabetes camps may help address these challenges by complementing routine care. They combine structured education, practical self-management training, and recreational activities while promoting peer support and reducing feelings of isolation. Camp formats vary widely in objectives (e.g., recreation vs. education), target groups (e.g., children, adolescents, or mixed-age groups), duration (e.g., from two days to several weeks), and size (e.g., 20 to over 500 participants) [4,5,6,7]. Studies have reported generally positive effects on diabetes-related distress, health-related quality of life, and metabolic control [8,9,10,11,12]. However, benefits are not consistent across all psychological domains. For instance, while improvements in diabetes-specific distress and overall well-being have been observed, effects on long-term anxiety reduction were less clear [13,14]. A recent systematic review and meta-analysis found benefits for diabetes knowledge and HbA1c, whereas improvements in quality of life and anxiety did not reach statistical significance [7]. The authors emphasized considerable heterogeneity in study designs, participant characteristics, camp programs, and outcome measures, underscoring the need for more targeted research including a longer observation period, various person-reported outcomes (PROs), and attention to underserved subgroups.

Despite these promising findings and distinct needs of this population [15], AYA remain underrepresented in diabetes camps [15]. As a result, data available on camp effectiveness specifically for AYA are limited. Importantly, the success of camp interventions may depend on how well they align with the participants’ backgrounds, challenges, and expectations. Understanding the characteristics of AYA who attend camps, and their wishes and needs, is essential to tailor programs and maximize engagement as well as psychosocial and clinical benefits.

This study therefore aimed to examine: (1) the characteristics of AYA attending diabetes camps; (2) their motivations and interests before camp; and (3) camp participation satisfaction, diabetes management, glycemic control, and PROs three months post-camp.

## 2. Materials and Methods

### 2.1. Study Design and Setting

This longitudinal observational study was conducted during *Camp D 2022*, Germany’s largest youth camp for individuals with type 1 diabetes. The camp was organized by a pharmaceutical company and supported by the German Diabetes Association (Deutsche Diabetes Gesellschaft: DDG). It took place over four days and was designed to strengthen AYA’s confidence in self-management, enhance practical diabetes management skills, and promote an active, self-determined lifestyle where the disease does not dominate or restrict daily life.

The camp program combined educational sessions, supervised leisure activities, and time for peer-to-peer exchange. Workshops covered age-relevant topics such as modern diabetes technologies, sexuality, career, and alcohol. Recreational and sports activities were also offered (e.g., cooking, singing, soccer, boxing, yoga, and water sports). Camp attendees were supervised in small groups by healthcare professionals and older peers. Individual counseling was also available when needed. A flat fee of €100 covered accommodation in tents, meals, and all activities.

### 2.2. Ethical Approval Statement

Written informed consent for participation in the study and data processing was obtained from all participants prior to the survey. As no adolescents were younger than 16 years, according to German law, no additional parental consent was required for minors aged 16 to 18. The study was reviewed and approved by the Ethics Committee of Hannover Medical School (No. 10373_BO_K_2022) and conducted in accordance with the Declaration of Helsinki.

### 2.3. Participants and Procedure

All 239 AYA registered for the camp were invited to participate in the study. Two weeks before the camp, participants received a link to an online questionnaire. The pre-camp survey collected demographic and clinical data, self-reported HbA1c, motivation for attending the camp, interest in specific camp topics, and baseline PROs using standardized questionnaires: mental well-being (World Health Organization Well-Being index: WHO-5) [16], diabetes burden (Problem Areas in Diabetes: PAID-5) [17], and anxiety (Generalized Anxiety Disorder: GAD-7) [18]. A follow-up online survey was sent three months after the camp including HbA1c, satisfaction, perceived effects of the camp, and the same PRO measures.

### 2.4. Measures

*Sociodemographic and clinical characteristics* included age, gender, education, migration background, living situation, comorbidities, psychotherapeutic treatment, diabetes duration, and type of insulin therapy. Participants reported their most recent HbA1c before and three months after the camp.

**Figure 1:**
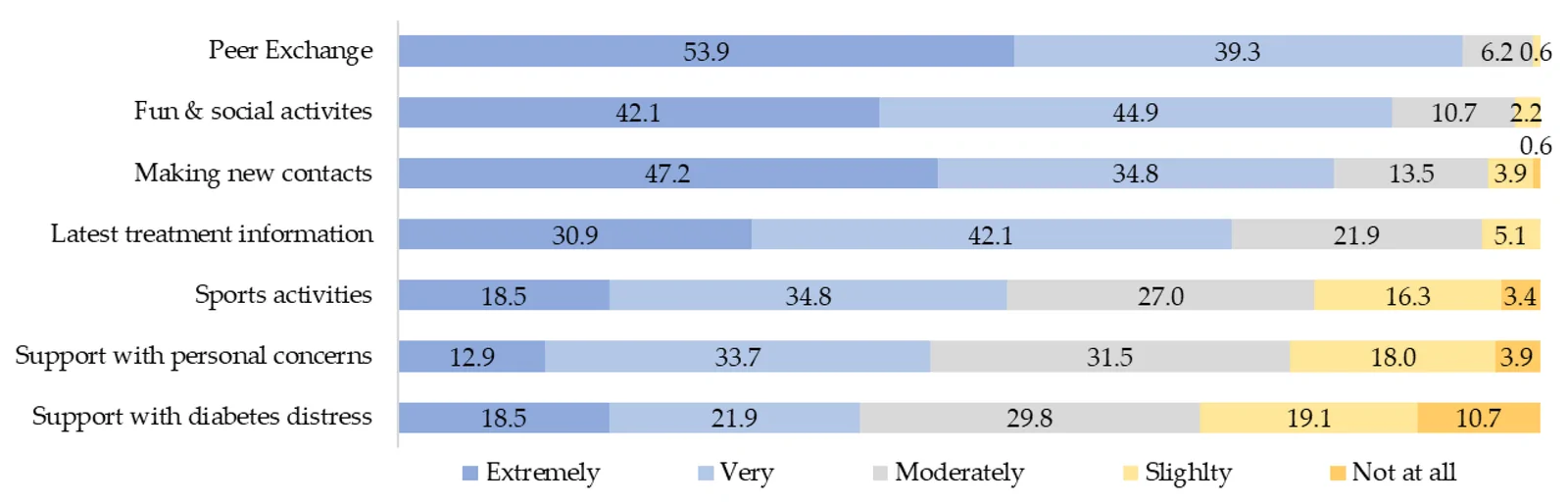
Frequency of primary motivations for enrolling in *Camp D* based on pre-camp survey responses (n = 178). Note. For analysis, positive ratings were defined as responses of “extremely” or “very” motivated and accounted for 93.3%, 87.1%, 82.0%, 73.0%, 53.4%, 46.6%, and 40.4% of responses across the seven respective items.

**Figure 2:**
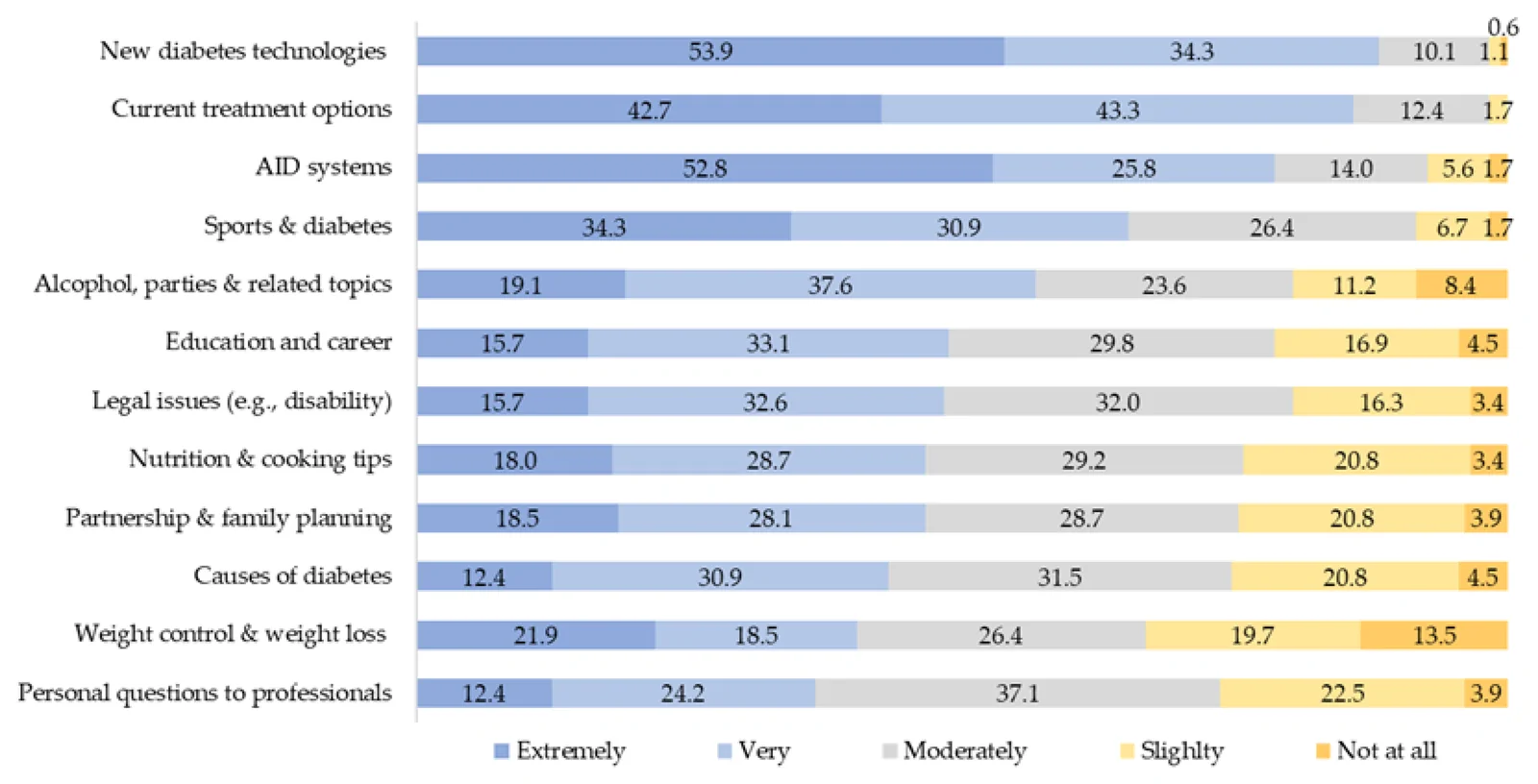
Frequency of interest in workshop topics offered during *Camp D* among participants based on pre-camp survey responses (n = 178). Note. For analysis, positive ratings were defined as responses of “extremely” or “very” interested and accounted for 88.2%, 86.0%, 78.7%, 65.2%, 56.7%, 48.9%, 48.3%, 46.6%, 46.6%, 43.3%, 40.4%, and 36.5% of responses across the respective items.

*Motivation to participate* was assessed pre-camp using seven items with the question “what motivated you to participate in the camp?” The items were rated using a 5-point Likert scale: “extremely”, “very”, “moderately”, “slightly” and “not at all”. See Figure 1 for motivation items. *Interest in 12 camp topics* was also assessed using a 5-point Likert scale (ranging from “extremely” to “not at all”). See Figure 2 for detailed topic items. For these variables, responses are presented using the original 5-point Likert scale for descriptive purposes. For analyses, responses were dichotomized into positive responses (“extremely” and “very”) and neutral-to-negative responses (“moderately”, “slightly”, and “not at all”).

*Satisfaction with participation* was evaluated three months after the camp using seven items rated from “very satisfied” to “very dissatisfied”. The items included overall satisfaction with the camp and content-specific aspects such as the information provided and the social opportunities. See Supplementary Figure S1 for the list.

*Perceived effects of participation* were assessed using four items addressing changes in self-treatment in everyday life, self-confidence in diabetes management, mental well-being, and motivation to manage diabetes. The items were rated using a 5-point Likert scale ranging from 1 (much worse) to 5 (much better). Participants were also asked whether they would recommend the camp to others.

*Diabetes-specific distress* (pre- and post-camp) was assessed using the PAID-5 [17], a five-item short version of the PAID questionnaire [19]. Scores range from 0 to 20, with higher values reflecting greater distress. A score ≥ 8 indicates significant diabetes distress.

*Mental well-being* was measured with the WHO-5 [16]. The five-item scale uses a Likert scale ranging from 0 to 5. Raw scores are then multiplied by four, resulting in a final score ranging from 0 (worst thinkable well-being) to 100 (best thinkable well-being). A score < 50 indicates poor well-being and ≤ 28 is indicative of likely depression [20].

*Anxiety* (pre- and post-camp) was assessed with the GAD-7 [18]. It is a seven-item measure of generalized anxiety symptoms with a total score ranging from 0 to 21. Higher scores are indicative of more severe anxiety symptoms, with a score ≥ 10 indicating clinically relevant anxiety.

### 2.5. Statistical Analysis

Data are described as frequencies, means (M) with standard deviations (±SD), and median (Mdn) with interquartile ranges (IQR). Group differences were examined using independent-samples *t*-tests or chi-squared tests. Pre- and post-camp comparisons were analyzed primarily using paired-samples *t*-tests. Wilcoxon signed-rank tests were conducted as sensitivity analyses. Changes in frequency were analyzed using McNemar’s test. Baseline demographic characteristics, camp motivations/interests, PROs, and glucose data were analyzed using all available baseline observations, with pairwise deletion applied for missing values. Analyses of change over time were restricted to participants with complete data at both assessment time points. Statistical significance was set at *p* < 0.05 (two-tailed). Analyses were performed using IBM SPSS 27.0 (SPSS Inc., Chicago, IL, USA). Because all eligible camp participants were invited to participate, no formal sample size calculation was performed. The sample size was determined by camp attendance; however, effect size estimates are reported to aid interpretation of the results.

## 3. Results

### 3.1. Participant Characteristics

Of 239 individuals registered for the camp, 178 completed the pre-camp survey (75% response rate). Characteristics of the sample are presented in Table 1. The sample had a mean age of 21.6 (±3.5) years (range: 16–29) and was predominantly female (69.7%). A migration background was reported by 17.4% of participants and was defined as having at least one parent born abroad. The sample was relatively well-educated with most holding a higher education qualification. The mean duration of diabetes was 10.8 (±5.9) years, and 59.7% met the recommended HbA1c target of <7.5%. Additional chronic comorbidities were reported by 31.6% (e.g., Hashimoto’s disease, celiac disease), and 25% were undergoing psychotherapy (e.g., for depression, an eating disorder, or anxiety).

Of the 178 participants enrolled at baseline, 71 completed the 3-month follow-up survey. To assess potential attrition bias, participants who completed the 3-month follow-up were compared with those who did not on baseline demographic, clinical, and psychosocial characteristics (see Supplementary Document). The two groups were comparable across all observed variables except HbA1c. Participants who completed the follow-up reported lower baseline HbA1c levels than those who did not (7.15 (±0.92)% vs. 7.63 (±1.14)%, respectively; mean difference = −0.48%, 95% CI (−0.83; −0.13), *t*(142) = −2.69, *p* = 0.008, Cohen’s *d* = −0.45, 95% CI (−0.789; −0.118)).

### 3.2. Motivations for Participation

The five-point Likert scale responses are presented in Figure 1. Dichotomized variable analyses revealed that connecting with others with T1D was the most commonly reported motivation for attending the camp. Having fun and establishing new contacts were the second and third most common motivations. Overall motivations were similar between male and female, but female participants were more likely to be motivated by peer exchange (96% vs. 86%; *χ*^2^ (1, 174) = 5.51, *p* = 0.019) and seeking support for diabetes-related emotional stress (47.6% vs. 22.0%; *χ*^2^ (1, 174) = 9.70, *p* = 0.002) (Supplementary Table S2).

*Exploratory subgroup analyses:* Motivation patterns were similar across age groups when stratified by age (≤21 vs. >21 years using the mean as cutoff). Participants with diabetes duration (≤10 years) were more interested in support with personal questions (56.3% vs. 37.4%; *χ*^2^ (1, 178) = 6.42, *p* = 0.011). Recently diagnosed participants with diabetes duration ≤ 2 years (n = 17) were less motivated by having fun/celebrating with others (70.6% vs. 88.8%; *χ*^2^ (1, 178) = 4.52, *p* = 0.033) but more motivated by up-to-date treatment information (94.1% vs. 70.8%; *χ*^2^ (1, 178) = 4.24, *p* = 0.039) and support with personal questions (76.5% vs. 43.5%; *χ*^2^ (1, 178) = 6.73, *p* = 0.010).

Motivations did not differ by migrant background or insulin pump use. Individuals already in psychological treatment were significantly more motivated by support with emotional distress (68.2% vs. 31.8%; *χ*^2^ (1, 176) = 18.05, *p* < 0.001).

### 3.3. Interests in Camp Offerings

Participants expressed the greatest interest in new diabetes technologies and current treatment options (Figure 2). Interests were largely similar between genders, although female participants were more interested in weight control and weight loss (46% vs. 28%; *χ*^2^ (1, 174) = 4.76, *p* = 0.029) (Supplementary Table S3).

*Exploratory subgroup analyses*: Older participants were significantly more interested in new technology (93.4% vs. 82.8%; *χ*^2^ (1, 178) = 4.85, *p* = 0.028), whereas younger participants were more interested in parties, alcohol and related topics (70.1% vs. 44%, *χ*^2^ (1, 178) = 12.40, *p* < 0.001), education, career start (60.9% vs. 37.4%, *χ*^2^ (1, 178) = 9.88, *p =* 0.002), and causes of diabetes (52.9% vs. 34.1%, *χ*^2^ (1, 178) = 6.41, *p =* 0.011).

Stratified by diabetes duration, participants with ≤ 10 years duration were more interested in sports and diabetes (77.0% vs. 53.8%, *χ*^2^ (1, 178) = 10.52, *p* < 0.001), nutrition and practical cooking tips (56.3% vs. 37.4%; *χ*^2^ (1, 178) = 6.42, *p* = 0.011), and personal questions to professionals (48.3% vs. 25.3%; *χ*^2^ (1, 178) = 10.15, *p* = 0.001).

Participants with a migrant background were more interested in causes of diabetes (61.3% vs. 39.5%; *χ*^2^(1, 178) = 4.97, *p* = 0.026). Insulin pump users (n = 132) and non-users (n = 46) had similar interests, although non-users showed greater interested in diabetes and parties, alcohol, and related topics (71.7% vs. 51.5%; *χ*^2^ (1, 178) = 5.684, *p* = 0.017).

### 3.4. Three-Month Post-Camp Satisfaction and Perceived Effects

Participants reported high satisfaction with the camp, with 97.2% reporting satisfaction. A detailed post-camp survey is presented in Supplementary Figure S1. Most participants reported a positive impact on their life, including improved motivation to manage diabetes (74.3%), self-treatment (64.3%), mental well-being (60.9%), and self-confidence in diabetes management (58.6%). Nearly all participants (98.6%) would recommend the camp to others. Within three months after the camp, 18 participants (25.4%) reported modifications in diabetes management, including adoption of new technology (n = 6) or changing diabetes care providers (n = 6).

### 3.5. Pre–Post Comparisons of PROs and HbA1c

*Diabetes distress (PAID-5)*. Baseline mean diabetes distress among all participants was 6.8 (±4.8), with 40.2% exceeding the high-burden score cutoff of ≥8 (Table 2). In the matched samples (n = 71), distress significantly decreased after the camp (mean change = −0.89, 95% CI (−1.55; −0.22), *t*(69) = −2.66, *p* = 0.010, Cohen’s *d* = −0.32, 95% CI (−0.557; −0.076), confirmed by a sensitivity test (*Z* = −2.49, *p* = 0.013). The proportion of AYA exceeding the distress cutoff decreased from 37% to 27%, but this was not statistically significant (*p* = 0.065).

*Well-being and depression (WHO-5).* Baseline mean well-being score was 52.0 (±21.3), with 45.6% scoring below 50 and 18.7% scoring ≤ 28. In the matched sample, well-being improved significantly after the camp (mean change = 4.63, 95% CI (1.13; 8.12), *t*(69) = 2.64, *p* = 0.010, *d* = 0.316, 95% CI (0.074; 0.555), confirmed by a sensitivity test (*Z* = −2.66, *p* = 0.008). The proportion meeting the depression cutoff declined from 18.6% to 9.9%, but it was not statistically significant (McNemar’s test, *p* = 0.109).

*Anxiety (GAD-7).* The baseline anxiety score was 7.8 (±5.2), with 31.0% scoring above the cutoff of ≥10. In the matched sample, a small reduction was observed, but the change was not statistically significant (mean difference = −0.31, 95% CI (−1.10; 0.47), *t*(69) = −0.80 *p* = 0.429, Cohen’s *d* = −0.095, 95% CI (−0.330; 0.140) (Table 2).

*HbA1c.* Mean baseline HbA1c was 7.43% (±1.08). For the paired analysis, no statistically significant change was noted in either the mean (mean difference = −0.04, 95% CI (−0.17; 0.09), *t*(59) = −0.58, *p* = 0.563, Cohen’s *d* = −0.075, 95% CI (−0.328; 0.179) or the proportion achieving HbA1c < 7.5% after the camp (Table 2).

*Sensitivity Analyses using Multiple Imputation*: Given the substantial loss to follow-up, multiple imputation was conducted as a sensitivity analysis to assess the robustness of the findings. The imputation model included baseline demographic, clinical, and psychosocial variables, and follow-up outcome measures. The results were consistent with those of the complete-case analysis (Table S1b).

## 4. Discussion

This study examined the characteristics, motivations, and PROs of AYA attending Germany’s largest diabetes camp. Several key findings emerged. First, the primary motivations for participation were peer exchange and emotional support. Second, AYA reported the greatest interest in new diabetes technologies and treatment topics. Third, participants reported high satisfaction and improvements in diabetes care, self-confidence, and motivation to manage diabetes. Finally, camp participation was associated with small but significant reductions in diabetes-specific distress and improvements in mental well-being but not with changes in general anxiety or HbA1c.

### 4.1. Characteristics of Camp Attendees, Motivation, and Interest

Camp participants represented a relatively selective group. A typical attendee was female, highly educated, and without a migrant background. This profile differs from studies in younger populations reporting a more balanced gender distribution [6,9]. However, the predominance of female and well-educated participants aligns with studies on health education and chronic-disease programs [21,22]. This likely reflects this group’s greater health engagement and motivation to participate in support programs.

Only 17.4% of participants reported a parental migrant background, compared with approximately 25% of the general German population [23]. Similar disparities have been documented in previous research on residential health camps for children and adolescents [24,25]. This underrepresentation may reflect structural, linguistic, or cultural barriers that limit access to programs outside routine care [26]. Moreover, financial barriers may have restricted participation. Although economically disadvantaged families could apply for fee waivers and travel support, communication challenges or cultural norms may have limited access. Financial barriers have also been reported as a challenge at camps in the United States [27]. Targeted recruitment strategies through diabetes clinics, culturally sensitive communication, simplified registration, and full financial support could enhance both diversity and accessibility among future camp participants [27].

The sample also showed a high burden of comorbid conditions and psychological challenges. One-third reported an additional chronic condition, and one-quarter received psychotherapeutic treatment. Moreover, 45.6% reported reduced well-being, with 18.7% indicating symptoms of depression, and 31.0% reported high anxiety levels (GAD-7 ≥ 10). Compared with recent surveys of AYA with T1D in Germany, where 21.2% reported reduced well-being and 19.0% met the anxiety threshold (GAD-7 ≥ 7), these rates appear higher, although this may partly reflect the overrepresentation of female participants [28,29]. It may also suggest that AYA experiencing greater burdens are more likely to enroll in camps. In contrast, glycemic control appeared to play a lesser role in participation decisions, with a mean HbA1c of 7.43% indicating relatively good metabolic control for this age group.

Together, these results highlight a potential gap in psychological support, emphasizing the importance of holistic services that integrate psychosocial care alongside medical and youth-specific content. Camps like this may therefore serve not only as educational and social experiences but also as valuable opportunities to support mental health and well-being among AYA.

Regarding motivation, peer connection was the strongest driver, supporting previous research suggesting that camps promote social integration and reduce isolation [5]. Diabetes technologies and therapy-related topics generated the greatest interest across all groups. Beyond these shared interests, the exploratory subgroup analyses highlighted diverse needs. For instance, female participants expressed greater interest in emotional support and weight management; those with shorter diabetes duration sought practical guidance; younger participants were more interested in topics related to parties and alcohol; and those receiving psychological treatment reported stronger interest in support for the psychological burden of diabetes. Tailoring camp programs to address diverse developmental and clinical needs, including targeted sessions for specific groups may enhance engagement and maximize their potential benefits.

### 4.2. Impact of Camp

Camp satisfaction was high, with participants reporting improved motivation, self-confidence, well-being, and self-management. These self-reported benefits were supported by standardized assessments showing small but statistically significant improvements in mental well-being three months post-camp. The proportion of individuals with critical scores in WHO-5 and PAID-5 was also reduced. This aligns with previous research demonstrating that diabetes camps can provide psychological benefits beyond routine medical care [8,9,10,11,12,30].

At baseline, participants reported high levels of diabetes distress, consistent with the literature on the emotional burden of daily diabetes management [3]. High stress levels among adolescents are often linked to continuous self-monitoring, fear of hypoglycemia and complications, and feelings of being alone with the condition or being “different” from peers [12,31]. Our findings suggest that camp participation may foster a sense of normality, social inclusion, and empowerment through peer interaction and psychosocial support [6,7,12]. Furthermore, approximately one in four participants reported adjusting their diabetes treatment within three months after the camp. This may reflect active engagement in treatment decision-making and increased self-management confidence.

Despite these benefits, no significant improvements were observed in general anxiety or HbA1c. While some studies have reported meaningful reductions in anxiety post-camp [32,33], our findings are consistent with prior research showing limited effects on generalized anxiety, even when significant improvements in mental well-being and diabetes distress were noted [6,7,9,13,14,24,34]. One possible explanation is that camps primarily address diabetes-specific concerns rather than generalized anxiety captured by the GAD-7. Diabetes-specific anxiety measures may have been more sensitive to changes related to camp participation. Similarly, no significant changes in HbA1c were observed. This is not surprising given that the camp’s workshops focused on mental health and empowering AYA to live fulfilled lives not constrained by T1D through engagement in age-appropriate activities rather than targeted HbA1c improvement.

### 4.3. Youth Care

By focusing on AYA transitioning into adulthood, this study contributes to understanding the role of alternative support models, such as youth camps, as complementary interventions in chronic disease management. Despite challenges in engaging AYA through traditional illness-related services, more than 200 AYA voluntarily registered for *Camp D*. Before the COVID-19 pandemic, attendance exceeded 300 participants. These figures illustrate the sustained appeal of recreationally framed support services for this population.

Beyond education, camps may provide psychological support, which is often inadequately addressed in conventional care [35]. Our findings emphasize the potential of complementary healthcare formats in addressing gaps in standard care. However, the limited diversity underscores the need for targeted recruitment strategies to engage underrepresented groups, including males, individuals with a migrant background, or those with lower socioeconomic status. Furthermore, a weekend intervention may be too brief to produce measurable changes in HbA1c or broader psychological outcomes. Follow-up approaches such as booster sessions, digital aftercare, or repeated camp participation may be necessary to maintain and enhance positive effects. Yet these measures are cost-intensive, and diabetes education outside formal training programs is not reimbursed by health insurance.

### 4.4. Limitations

Strengths of this study include a relatively large baseline sample, pre- and post-camp assessments, use of standardized questionnaires, and a focus on AYA, an often-underserved group. However, the study has some limitations. First, reliance on self-reported outcomes may have introduced reporting biases. Second, substantial loss to follow-up represents an important limitation of the study. Only 71 of the 178 baseline participants completed the 3-month assessment, raising the possibility of attrition and selection bias. Comparison of baseline characteristics indicated that participants who completed follow-up differed from non-responders in baseline HbA1c. This may reflect response bias, as individuals who were more engaged in diabetes management may have been more likely to respond to the follow-up assessment. Consequently, the complete-case paired analyses should be interpreted as estimates among participants who completed the follow-up and may not fully generalize to the original baseline cohort. Although a sensitivity analysis using multiple imputation produced similar results, missingness cannot be ruled out as a source of bias. Third, the absence of a control group limits causal interpretation. Finally, exploratory subgroup analyses should be interpreted with caution due to the number of comparisons and small sample sizes.

## 5. Conclusions

Participation in the youth camp was associated with high satisfaction, perceived improvements in diabetes self-management, confidence, and motivation, and reduced diabetes distress among AYA. Youth camps may therefore represent a promising complement to outpatient care during this challenging life stage. At the same time, the demographic profile of participants highlights inequities in access, emphasizing the need for broader outreach and targeted recruitment to ensure diverse representation. Understanding participant characteristics, motivations, and interests may help inform targeted recruitment strategies and the tailoring of camp content to meet their needs.

## Figures and Tables

**Table 1:** Baseline sample characteristics.

Variable	N	All Participants (Baseline, N = 178)
Age, years	178	21.6 ± 3.5
Gender, female	178	124 (69.7%)
Migrant background, at least one parent born abroad	178	31 (17.4%)
Living conditions	178	
Living with both parents/one parent		91 (51.1%)
Living with a partner		32 (18.0%)
Living in a shared accommodation		21 (11.8%)
Living alone		32 (18.0%)
Different living situation		2 (1.1%)
Education	178	
Still attending school		32 (18.0%)
Intermediate/advanced secondary education		60 (33.7%)
General higher education, ≥12 years		86 (48.3%)
Diabetes duration, years	178	10.8 (±5.9)
Use of insulin pump (CSII)	178	132 (74.2%)
Use of continuous glucose monitoring (CGM)	178	159 (89.3%)
Use of automated insulin dosing (AID)	178	61 (34.3%)
HbA1c in % (self-reported)	144	7.43 (±1.08), Mdn = 7.3 (IQR 6.73–7.98)
HbA1c, % < 7.5		86 (59.7%)
Comorbidities, yes	177	56 (31.6%)
Psychological treatment, yes	176	44 (25.0%)

Note. Percentages were calculated excluding missing values. Missing data reflect unreported responses.

**Table 2:** Comparison of psychological parameters and HbA1c before and three months after the camp for all study participants and paired participants.

	N	Pre-Camp, N = 178	Paired n	Pre-Camp	Post-Camp	*p*	Cohen’s *d*
PAID-5 (diabetes distress)	169		70				
M (±SD); Mdn (IQR)		6.8 (±4.8),6 (3–10)		6.5 (±4.6); 6 (3–10)	5.7 (±4.0); 5 (2.75–8.25)	0.010	−0.317
High distress ≥ 8, n (%)		68 (40.2%)		26 (37.1%)	19 (27.1%)	0.065	
WHO-5 (mental well-being)	171		70				
M (±SD); Mdn (IQR)		52.0 (±21.3); 52 (36–68)		52.3 (±20.5); 52 (43–68)	57.0 (±19.7); 60 (40–72)	0.010	0.316
Depression, ≤28, n (%)		32 (18.7%)		13 (18.6%)	7 (10.0%)	0.109	
GAD-7 (anxiety)	171		70				
M (±SD); Mdn (IQR)		7.8 (±5.2); 7 (4–10)		7.6 (±5.5); 6 (3–11)	7.3 (±5.2); 7 (3–10.25)	0.429	−0.095
High anxiety, ≥10, n (%)		53 (31%)		20 (28.6%)	18 (25.7%)	0.687	
HbA1c, %	144		60				
M (±SD); Mdn (IQR)		7.43 (±1.08); 7.3 (6.73–7.98)		7.15 (±0.92); 7.25 (6.43–7.75)	7.11 (±0.92); 7.20 (6.53–7.60)	0.563	−0.075
<7.5%		86 (59.7%)		39 (65.0%)	41 (68.3%)	0.774	

Note. PAID, problem areas in diabetes; WHO, World Health Organization; GAD, generalized anxiety disorder. *p* values are based on paired *t*-test and McNemar’s test results. Percentages were calculated excluding missing values. Missing data reflect unreported responses.

## Data Availability

The data presented in this study are available on request from the corresponding author. The data are not publicly available due to privacy and ethical reasons.

## Supplementary Materials

The following supporting information can be downloaded at: https://www.mdpi.com/article/10.3390/children13101386/s1, Figure S1: Satisfaction with Camp-D among participants in the post-camp survey (n = 71), Table S1a: Baseline characteristics of participants with and without 3-month follow-up data, Table S1b: Comparison of psychological parameters and HbA1c before and three months after the camp using multiple imputation, Table S2: Motivation to participate in Camp-D by groups, Table S3: Interests in camp topic areas by groups.

## Abbreviations

(AID) automated insulin dosing, (AYA) adolescents and young adults, (CGM) continuous glucose monitoring, (COVID-19) coronavirus disease 2019, (CSII) continuous subcutaneous insulin infusion, (DDG) Deutsche Diabetes Gesellschaft (German Diabetes Association), (GAD-7) Generalized Anxiety Disorder Scale, (HbA1c) glycated hemoglobin A1c, (PAID-5) Problem Areas in Diabetes Scale 5, (PRO) person-reported outcome, (WHO-5) World Health Organization Wellbeing Index, (T1D) type 1 diabetes
