# Supplementary material for: Insights from Germany’s Largest Diabetes Camp for Young People: Who Attends, Why, and What Do They Gain?
Source: Children (Basel). 2026 Oct 12;13(10):1386. doi: 10.3390/children13101386 (PMC13703593; doi:10.3390/children13101386)
Supplement: Supplementary file 1 [file children-13-01386-s001.zip › children-4566779-supplementary.pdf]

Supplementary Document

Figure S1. Satisfaction with Camp-D among participants in the post-camp survey (n = 71)

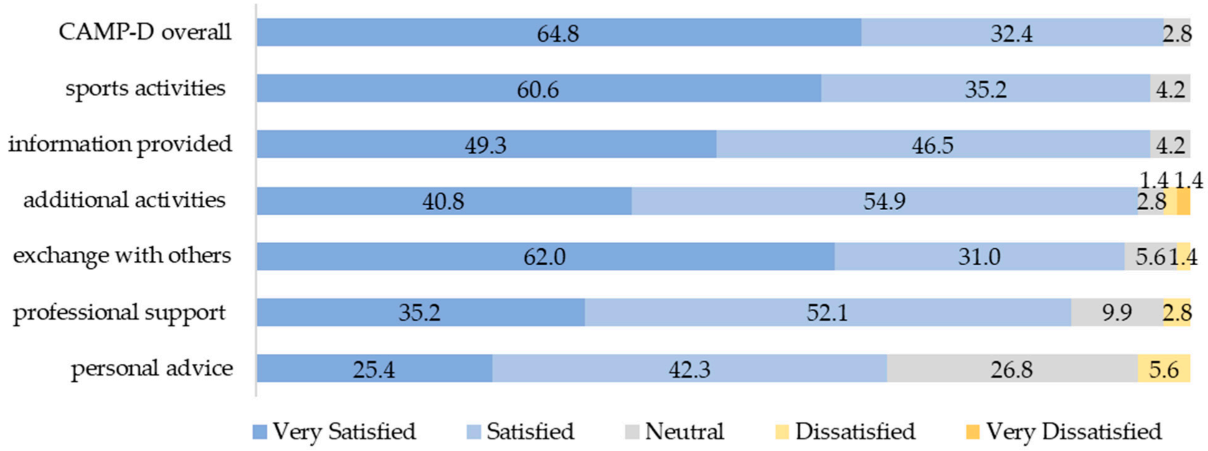

Note. Positive responses (Very Satisfied or Satisfied) accounted for 97.2%, 95.8%, 95.8%, 95.7%, 93.0%, 87.3%, and 67.7% of responses across the respective items.

Table S1a. Baseline characteristics of participants with and without 3-month follow-up data

	Completed both surveys		Pre-camp survey only		
Variables	n	M (SD) or N (%)	n	M (SD) or N (%)	*p*
Age, years	71	21.7 ± 3.3	107	21.6 ± 3.7	
Gender, female	71	50 (70.4 %)	107	74 (69.2 %)	
Migrant background, at least one parent born abroad	71	9 (12.6%)	107	22 (20.6%)	
Education	71		107		
Still attending school		13 (18.3 %)		19 (17.8 %)	
Intermediate/Advanced Secondary Education		22 (31.0 %)		38 (35.4 %)	
General Higher Education, ≥ 12 years completed		36 (50.7 %)		50 (46.7 %)	
Diabetes duration, years	71	10.4 (± 6.0)	107	11.1 (± 5.8)	
Use of insulin pump (CSII)	71	55 (77.5 %)	107	77 (72.0 %)	
Use of continuous glucose monitoring (CGM)	71	65 (91.5 %)	107	94 (87.9 %)	
Use of Automated Insulin Dosing (AID)	71	25 (35.2 %)	107	36 (33.6 %)	
HbA1c in % (self-reported)	60	7.15 % (± .92), Mdn = 7.25 (IQR 6.43-7.75)	84	7.62 (±1.14), Mdn = 7.40 (IQR 6.90-8.20)	.008
HbA1c, % < 7.5		39 (65.0%)		47 (56.0%)	
Comorbidities, yes	71	18 (25.4 %)	106	38 (35.8 %)	
Psychological treatment, yes	71	18 (25.4 %)	105	26 (24.8 %)	
PAID-5	70	6.54 (4.57)	101	7.03 (4.96)	
WHO-5	70	52.3 (20.5)	101	51.8 (22.0)	
GAD-7	70	7.57 (5.47)	101	7.89 (4.99)	

Note. Percentages were calculated excluding missing values. Missing data reflect unreported responses.

Table S1b. Comparison of psychological parameters and HbA1c before and three months after the camp using multiple imputation.

Pairs: Pre-Post	MEAN	SE of Mean	95% CI; Lower	Upper	t	df	p
PAID-5	1.060	.4070	.255	1.866	2.606	120	.010
WHO-5	-5.154	1.912	-8.928	-1.380	-2.695	164	.008
GAD-7	.6070	.4665	-.315	1.529	1.301	128	.196
HbA1c	.1139	.0856	-.055	.283	1.330	111	.186

Table S2. Motivation to participate in Camp-D by groups

MOTIVATION					
**BY Gender**					
Female, n = 124	Frequency	Percent	Male, n = 50	Frequency	Percent
Exchange with peers *	119	96	Having fun/celebrating with others	47	94
Having fun/celebrating with others	106	85.5	Exchange with peers *	43	86
Making new contacts	104	83.9	Making new contacts	38	76
Up-to-date information on treatment	95	76.6	Doing sports	32	64
Doing sports	62	50	Up-to-date information on treatment	32	64
Support with emotional stress caused by diabetes **	59	47.6	Support with personal questions	25	50
Support with personal questions	56	45.2	Support with emotional stress caused by diabetes **	11	22
**BY Age**					
Age 16-21, n = 87	Frequency	Percent	22+, n = 91	Frequency	Percent
Exchange with peers	82	94.3	Exchange with peers	84	92.3
Having fun/celebrating with others	75	86.2	Having fun/celebrating with others	80	87.9
Making new contacts	68	78.2	Making new contacts	78	85.7
Up-to-date information on treatment	61	70.1	Up-to-date information on treatment	69	75.8
Doing sports	52	59.8	Support with personal questions	46	50.5
Support with personal questions	37	42.5	Doing sports	43	47.3
Support with emotional stress caused by diabetes	34	39.1	Support with emotional stress caused by diabetes	38	41.8
**BY Diabetes Duration**					
≤ 10 years, N=87	Frequency	Percent	11+, n = 91	Frequency	Percent
Exchange with peers	82	94.3	Exchange with peers	84	92.3
Having fun/celebrating with others	74	85.1	Having fun/celebrating with others	81	89.0
Making new contacts	70	80.5	Making new contacts	76	83.5
Up-to-date information on treatment	66	75.9	Up-to-date information on treatment	64	70.3
Doing sports	50	57.5	Doing sports	45	49.5
Support with personal questions*	49	56.3	Support with personal questions*	34	37.4
Support with emotional stress caused by diabetes	39	44.8	Support with emotional stress caused by diabetes	33	36.3

BY Parental migrant background					
German Born, n = 147	Frequency	Percent	Non-German Born Parent, n = 31	Frequency	Percent
Exchange with peers	136	92.5	Exchange with peers	30	96.8
Having fun/celebrating with others	130	88.4	Making new contacts	27	87.1
Making new contacts	119	81.0	Having fun/celebrating with others	25	80.6
Up-to-date information on diabetes treatment	105	71.4	Up-to-date information on diabetes treatment	25	80.6
Doing sports	81	55.1	Support with personal questions	17	54.8
Support with personal questions	66	44.9	Support with emotional stress caused by diabetes	16	51.6
Support with emotional stress caused by diabetes	56	38.1	Doing sports	14	45.2
**BY Insulin delivery**					
Pen/ needle, n = 46	Frequency	Percent	Pump, n = 132	Frequency	Percent
Exchange with peers	44	95.7	Exchange with peers	122	92.4
Making new contacts	39	84.8	Having fun/celebrating with others	118	89.4
Having fun/celebrating with others	37	80.4	Making new contacts	107	81.1
Up-to-date information on diabetes treatment	37	80.4	Up-to-date information on diabetes treatment	93	70.5
Doing sports	25	54.3	Doing sports	70	53.0
Support with personal questions	23	50.0	Support with personal questions	60	45.5
Support with emotional stress caused by diabetes	17	37.0	Support with emotional stress caused by diabetes	55	41.7

Note. * $p < .05$, ** < .01 according to Chi-squared test.

Table S3. Interests in camp topic areas by groups

INTERESTS					
**BY Gender**					
Female, n = 124	Frequency	Percent	Male, n = 50	Frequency	Percent
Current treatment options	109	87.9	New diabetes technologies (pumps, sensors, etc)	45	90.0
New diabetes technologies (pumps, sensors, etc)	109	87.9	Current treatment options	41	82.0
AID systems (artificial pancreas)	97	78.2	AID systems (artificial pancreas)	41	82.0
Sports and diabetes	80	64.5	Sports and diabetes	32	64.0
Parties, alcohol, and related topics	66	53.2	Parties, alcohol, and related topics	32	64.0
Education, studies, and career start	65	52.4	Legal aspects (driver's license, disability, etc)	24	48.0
Nutrition and practical cooking tips	59	47.6	Causes of diabetes	22	44.0
Partnership and family planning	59	47.6	Partnership and family planning	22	44.0
Legal aspects (driver's license, disability, etc)	59	47.6	Education, studies, and career start	21	42.0
Weight control, weight loss *	57	46	Personal questions to professionals	21	42.0
Causes of diabetes	54	43.5	Nutrition and practical cooking tips	20	40.0
Personal questions to professionals	43	34.7	Weight control, weight loss *	14	28.0
**BY Age**					
Age 16-21, n = 87	Frequency	Percent	22+, n = 91	Frequency	Percent
Current treatment options	74	85.1	New diabetes technologies (pumps, sensors, etc)*	85	93.4
New diabetes technologies (pumps, sensors, etc)*	72	82.8	Current treatment options	79	86.8
AID systems (artificial pancreas)	65	74.7	AID systems (artificial pancreas)	75	82.4
Parties, alcohol, and related topics ***	61	70.1	Sports and diabetes	56	61.5
Sports and diabetes	60	69.0	Partnership and family planning	46	50.5
Education, studies, and career start**	53	60.9	Nutrition and practical cooking tips	43	47.3
Causes of diabetes *	46	52.9	Legal aspects (driver's license, disability, etc)	41	45.1
Legal aspects (driver's license, disability, etc)	45	51.7	Parties, alcohol, and related topics***	40	44.0
Nutrition and practical cooking tips	40	46.0	Weight control, weight loss	35	38.5
Weight control, weight loss	37	42.5	Education, studies, and career start**	34	37.4
Partnership and family planning	37	42.5	Personal questions to professionals	33	36.3
Personal questions to professionals	32	36.8	Causes of diabetes *	31	34.1

**BY Diabetes Duration**					
≤ 10 years, N=87	Frequency	Percent	11+, n = 91	Frequency	Percent
New diabetes technologies (pumps, sensors, etc)	79	90.8	Current treatment options	78	85.7
Current treatment options	75	86.2	New diabetes technologies (pumps, sensors, etc)	78	85.7
Sports and diabetes**	67	77.0	AID systems (artificial pancreas)	76	83.5
AID systems (artificial pancreas)	64	73.6	Sports and diabetes**	49	53.8
Parties, alcohol, and related topics	53	60.9	Parties, alcohol, and related topics	48	52.7
Nutrition and practical cooking tips*	49	56.3	Partnership and family planning	44	48.4
Education, studies, and career start	46	52.9	Legal aspects (driver's license, disability, etc)	42	46.2
Legal aspects (driver's license, disability, etc)	44	50.6	Education, studies, and career start	41	45.1
Personal questions to professionals**	42	48.3	Causes of diabetes	38	41.8
Causes of diabetes	39	44.8	Nutrition and practical cooking tips*	34	37.4
Weight control, weight loss	39	44.8	Weight control, weight loss	33	36.3
Partnership and family planning	39	44.8	Personal questions to professionals**	23	25.3
**BY Parental migrant background**					
German Born, n = 147	Frequency	Percent	Non-German Born Parent, n = 31	Frequency	Percent
New technologies (pumps, sensors)	128	87.1	New technologies (pumps, sensors)	29	93.5
Current treatment options	125	85.0	Current treatment options	28	90.3
AID systems (artificial pancreas)	115	78.2	AID systems (artificial pancreas)	25	80.6
Sports and diabetes	98	66.7	Causes of diabetes	19	61.3
Parties, alcohol, and related topics	88	59.9	Sports and diabetes	18	58.1
Education, studies, and career start	72	49.0	Legal aspects (e.g., driver's license, disability status)	17	54.8
Nutrition and practical cooking tips	71	48.3	Weight control and weight loss	15	48.4
Partnership and family planning	70	47.6	Education, studies, and career start	15	48.4
Legal aspects (e.g., driver's license, disability status)	69	46.9	Personal questions to professionals	14	45.2
Causes of diabetes	58	39.5	Partnership and family planning	13	41.9
Weight control and weight loss	57	38.8	Parties, alcohol, and related topics	13	41.9
Personal questions to professionals	51	34.7	Nutrition and practical cooking tips	12	38.7
**BY Insulin delivery**					
Pen/ needle, n = 46	Frequency	Percent	Pump, n = 132	Frequency	Percent
New technologies (pumps, sensors)	43	93.5	New technologies (pumps, sensors)	114	86.4

Current treatment options	42	91.3	Current treatment options	111	84.1
AID systems (artificial pancreas)	35	76.1	AID systems (artificial pancreas)	105	79.5
Sports and diabetes	33	71.7	Sports and diabetes	83	62.9
Parties, alcohol, and related topics *	33	71.7	Parties, alcohol, and related topics *	68	51.5
Nutrition and practical cooking tips	27	58.7	Education, studies, and career start	65	49.2
Weight control and weight loss	23	50.0	Legal aspects (e.g., driver's license, disability status)	64	48.5
Partnership and family planning	22	47.8	Partnership and family planning	61	46.2
Legal aspects (e.g., driver's license, disability status)	22	47.8	Causes of diabetes	56	42.4
Education, studies, and career start	22	47.8	Nutrition and practical cooking tips	56	42.4
Causes of diabetes	21	45.7	Weight control and weight loss	49	37.1
Personal questions to professionals	16	34.8	Personal questions to professionals	49	37.1

Note. * $p < .05$, ** < .01, *** < .001 according to Chi-squared test.
